# Supplementary material for: The genome sequence of the Shy Cosmet Moth, Limnaecia phragmitella Stainton, 1851 (Lepidoptera: Cosmopterigidae)
Source: Wellcome Open Res. 2025 Sep 15;10:509. [Version 1] doi: 10.12688/wellcomeopenres.24881.1 (PMC12552839; doi:10.12688/wellcomeopenres.24881.1)
Supplement: Peer review history

# Reviewer report: Reviewer response for version 1

**DOI:** 10.21956/wellcomeopenres.27406.r135613

**Published:** 2025-10-24

**Authors:** Panagiotis Ioannidis

**Affiliations:** 1 Foundation for Research & Technology - Hellas, Crete, Greece

This manuscript describes the sequencing and assembly of Limnaecia phragmitella, a lepidopteran insect.

 The methodology is identical to the one used by other DToL projects. All assembly metrics (N50, completeness, QV, BUSCO, blobplot) show that the assembly quality is very good.

 ​​​​​​Recently, I have come to realize that providing a genome assembly without an accompanying gene set is of limited to most scientists; very few are either interest in the genome sequence per se, or know how to perform gene prediction.

 Related to the above, the authors mention that "The genome will be annotated using available RNA-Seq data...". What are these RNAseq data? Are they from the same organism? Because if they're not I would guess that the resulting gene set would be of relatively low quality.

Are sufficient details of methods and materials provided to allow replication by others?

Yes

Is the rationale for creating the dataset(s) clearly described?

Yes

Are the datasets clearly presented in a useable and accessible format?

Yes

Are the protocols appropriate and is the work technically sound?

Yes

Reviewer Expertise:

insect genomics; bioinformatics

I confirm that I have read this submission and believe that I have an appropriate level of expertise to confirm that it is of an acceptable scientific standard.

# Reviewer report: Reviewer response for version 1

**DOI:** 10.21956/wellcomeopenres.27406.r135610

**Published:** 2025-10-24

**Authors:** Arun Arumugaperumal

**Affiliations:** 1 Department of Biotechnology, Rajalakshmi Engineering College, Thandalam, Chennai, Tamil NaduIndia, 602105, India

The data note describes the genome sequencing of the Shy Cosmet Moth, *Limnaecia phragmitella*. The authors have used long-read sequencing and Hi-C mapping to obtain a high-quality genome sequence. The assembly reported here is of size 539.22 Mb spread among 30 chromosomes. Since a male specimen has been used for sequencing, the authors have captured only the Z sex chromosome. A female specimen could have been used. The mitogenome has also been presented of size 15.26 kb. The annotations were yet to be released in Ensembl. The genome sequence achieved a EBP summary metric of 5.C.Q55. However, the contig N50 value is less compared to the benchmark. The BUSCO completeness percentage of 98.5 indicates that the genome is near-complete with respect to sequences in the database. 

 In the introduction part, check the phrase 'The early instar larvae mines in the leaf sheaf..'. Is it sheath or sheaf?

 The article can be indexed.

Are sufficient details of methods and materials provided to allow replication by others?

Yes

Is the rationale for creating the dataset(s) clearly described?

Yes

Are the datasets clearly presented in a useable and accessible format?

Yes

Are the protocols appropriate and is the work technically sound?

Yes

Reviewer Expertise:

Bioinformatics; Genomics

I confirm that I have read this submission and believe that I have an appropriate level of expertise to confirm that it is of an acceptable scientific standard.
